# Association of preserved ratio impaired spirometry with mortality and cardiovascular diseases: a systematic review and meta-analysis

**DOI:** 10.1186/s13643-024-02549-6

**Published:** 2024-07-06

**Authors:** Mengya Li, Mengxin Chen, Yang Li, Zhiguang Liu, Xiaocong Li, Xinyue Lang, Bo Hu, Wei Li

**Affiliations:** 1 National Center for Cardiovascular Diseases, National Clinical Research Center for Cardiovascular Diseases, Chinese Academy of Medical Sciences Fuwai Hospital, Peking Union Medical College, Beijing, 102308 China; 2 Interventional Center of Valvular Heart Disease, Beijing Anzhen Hospital, Capital Medical University, Beijing, China; 3 Department of Pharmacy and Clinical Trial Unit, Beijing Anzhen Hospital, Capital Medical University, Beijing, China

**Keywords:** Cardiovascular disease, Lung function, Mortality, Preserved ratio impaired spirometry (PRISm), Risk, Spirometry classification

## Abstract

**Background:**

Preserved ratio impaired spirometry (PRISm) is a type of abnormal lung function. PRISm and mortality have been explored in several studies, but a comprehensive evaluation of the associations is limited. The current study aims to conduct a systematic review and meta-analysis in order to investigate the mortality and cardiovascular diseases in patients with PRISm.

**Methods:**

PubMed, Embase, and Web of Science databases, as well as gray literature sources, were searched for relevant studies published up to 7 September 2023 without language restrictions. This review included all published observational cohort studies that investigated the association of PRISm with mortality in the general population, as well as subgroup analyses in smokers and pre-bronchodilation spirometry studies. The outcomes of interest were all-cause mortality, cardiovascular mortality, and respiratory-related mortality. The Newcastle–Ottawa scale assessed study quality. Sensitivity and subgroup analyses explored heterogeneity and robustness. Publication bias was assessed with Egger’s and Begg’s tests.

**Results:**

Overall, eight studies were included in this meta-analysis. The pooled HR was 1.60 (95% *CI*, 1.48–1.74) for all-cause mortality, 1.68 (95% *CI*, 1.46–1.94) for CVD mortality, and 3.09 (95% *CI*, 1.42–6.71) for respiratory-related mortality in PRISm group compared to normal group. In the subgroup analysis, participants with PRISm had a higher effect (*HR*, 2.11; 95% *CI*, 1.74–2.54) on all-cause mortality among smokers relative to participants with normal spirometry. Furthermore, the association between PRISm and mortality risk was consistent across several sensitivity analyses.

**Conclusions:**

People with PRISm were associated with an increased risk of all-cause mortality, CVD mortality, and respiratory-related mortality as compared to those with normal lung function in the general population.

**Systematic review registration:**

PROSPERO CRD42023426872.

**Supplementary Information:**

The online version contains supplementary material available at 10.1186/s13643-024-02549-6.

## Background

Preserved ratio impaired spirometry (PRISm) is a special type of lung function abnormality that has been previously referred to as Global Initiative for Chronic Obstructive Lung Disease (GOLD)-unclassified. It is defined as a condition where the ratio of the forced expiratory volume in one second (FEV1) to the forced vital capacity (FVC) is normal (FEV1/FVC ≥ 0.7 after bronchodilation) but the abnormal spirometry (FEV1 < 80% predicted value after bronchodilation) [1].

PRISm is a lung function abnormality, with reported prevalence rates ranging from 4 to 48% [2]. The variation in prevalence rates may be related to gender, race, geographical location, smoking, risk factors, and different predicted value selection. Furthermore, several longitudinal cohort studies have shown an association between PRISm and an increased risk of all-cause mortality, as well as the incidence and mortality of cardiovascular and respiratory-related diseases [2–9]. Due to its high prevalence in the population and the risk of adverse health outcomes, research related to PRISm has increased. However, existing studies investigating the association between PRISm and adverse outcomes such as all-cause mortality and cardiovascular disease (CVD) mortality have mainly used data from a few developed countries, including the United States, Canada, the UK, Denmark, the Netherlands, Japan, and South Korea. Therefore, the conclusions of these studies cannot be directly extrapolated to a global population. Moreover, there is significant variation in the estimates of whether PRISm is an independent risk factor for CVD mortality and its hazard ratio (HR) among different studies. While a recent meta-analyses [10] have explored this association, our study seeks to extend this research by including more latest published literature with wider geographical areas, subgroup analysis among smoking population and pre-bronchodilation spirometry studies, and more sensitivities and quality assessment to make the study more comprehensive. Therefore, we conducted a systematic review and meta-analysis to summarize the existing evidence on the association between PRISm and adverse outcomes including all-cause mortality, CVD mortality, and respiratory-related mortality.

## Methods

### Literature search and selection criteria

For this meta-analysis, PubMed, Embase, and Web of Science databases, as well as gray literature sources, were searched for relevant studies published up to 7 September 2023 without language restrictions, supplemented by manual searches of the references of retrieved articles. In addition, the first 10 pages of the Google Scholar search engine were manually searched for gray literature. The searches were performed by one author and then double checked by other authors. The search strategy we used involved certain keywords such as “preserved ratio-impaired spirometry,” “PRISm spirometry,” “restrictive spirometry,” “restrictive lung disease,” “restrictive pulmonary disease,” “restrictive lung patterns,” “restrictive lung defect,” “restrictive pulmonary defect,” “restrictive lung function,” “restrictive pulmonary function,” “low lung function,” “unclassified spirometry,” “nonspecific spirometry,” “global initiative for chronic obstructive lung disease unclassified,” “GOLD-unclassified,” and “GOLD-U.” A detailed description of this strategy for each database is given in Additional file 1: Table S1. This study was conducted according to the Preferred Reporting Items for Systematic Reviews and Meta-Analyses (PRISMA) guideline [11] as outlined in Additional file 1: Table S2 and was registered with PROSPERO (CRD42023426872).

### Study selection

**Fig. 1:**
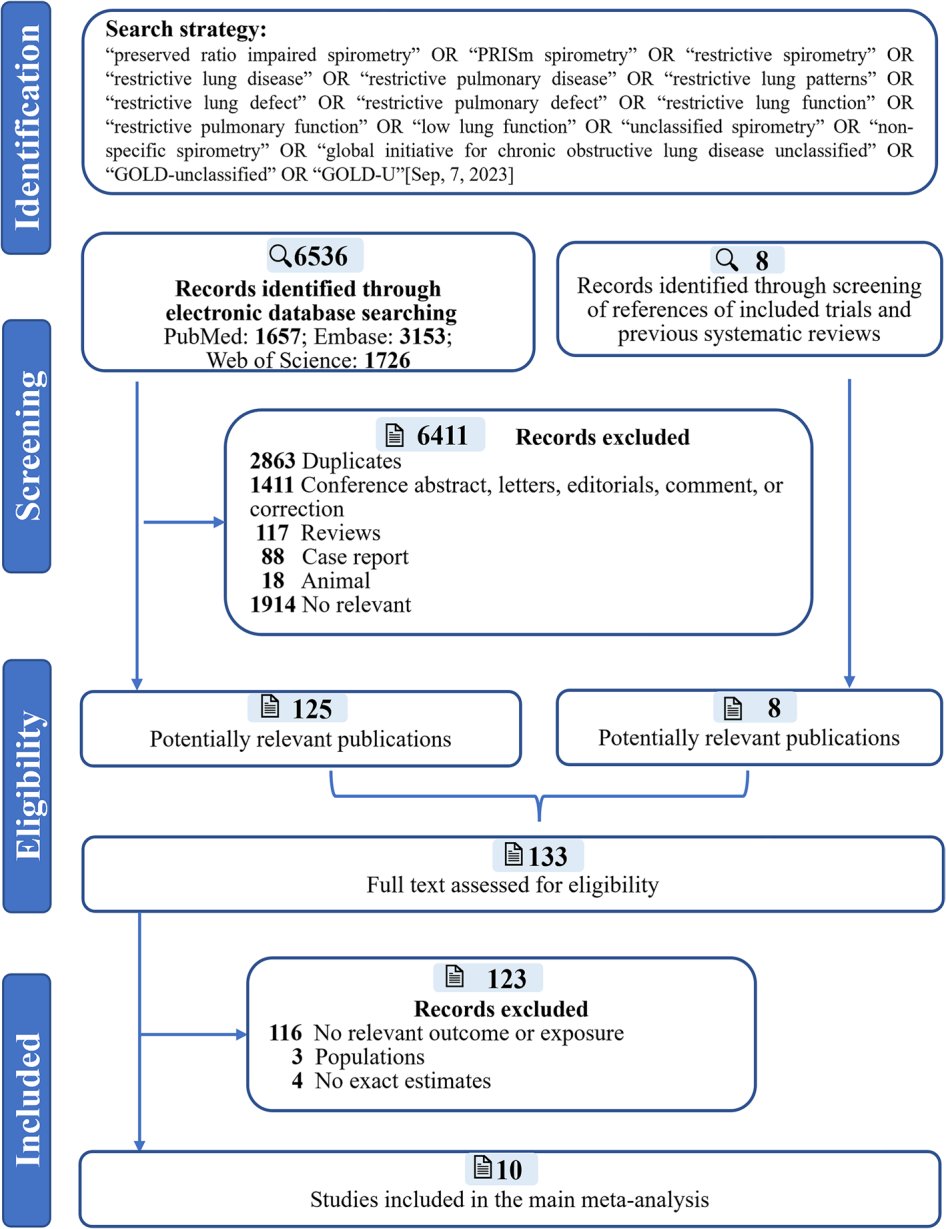
Flowchart of the selection of studies included in the meta-analysis

All the articles identified through the electronic and manual searches were exported to EndNote, version 19, and any duplicates were removed. Two authors independently screened the title and abstract of the articles and excluded those that were irrelevant. The full text of each potentially eligible reference was further reviewed to identify characteristics of the study design, objective, and associations reported. We used the following inclusion criteria: (1) study types restricted to cohort studies in the general population, (2) studies with pre- or post-bronchodilation FEV1/FVC ≥ 0.7 or lower limit of normal (LLN) and further divided them into PRISm and healthy controls based on whether *FEV1* < 80% predicted or LLN, and (3) quantitative analysis with HR of main results and 95% CI or enough information to calculate. The exclusion criteria included the following: (1) reviews, letters, case report, conference presentations, abstracts, editorials, expert opinions, and animal or fundamental studies; however, their references were systematically searched, (2) participants with special pathological conditions (type 2 diabetes), and (3) duplicated cohort population. Any discrepancies were resolved through discussion among the reviewers and consultation with a senior scholar. A detailed description of the search and selection strategy is presented in Fig. 1.

### Main exposure and outcomes

Lung function was assessed using pre-bronchodilation spirometry examination except Wan (2018) and Perez-Padilla (2023) which used post-bronchodilation spirometry [12]. We focused on the exposure: PRISm was defined as having a pre- or post-bronchodilation FEV1: FVC ratio greater than or equal to 0.7 and an FEV1 less than 80% predicted, and normal spirometry was defined as an FEV1: FVC ratio greater than or equal to 0.7 and an FEV1 greater than or equal to 80% predicted. In sensitivity analyses of several studies, a lower limit of normal (LLN) thresholds was used to define lung function categories [7]. PRISm-LLN was defined as having an FEV1: FVC ratio greater than or equal to LLN and an FEV1 less than LLN, and normal spirometry-LLN was defined as an FEV1: FVC ratio greater than or equal to LLN and an FEV1 greater than or equal to LLN.

The outcomes of interest were all-cause mortality, CVD mortality, and respiratory-related mortality. Effect sizes were expressed as HRs and their 95%CIs calculated for each study, based on the risk of mortality in individuals with PRISm versus normal spirometry individuals.

### Data extraction

One author extracted study details and relevant results from observational cohort studies that met inclusion criteria, and another author validated them independently. Inconsistencies between the two reviewers were discussed for clarification and agreement on final reporting. For each study, we extracted the following information: (1) the basic information about the study: title, the lead investigator, country, publication date, journal, and the name of cohort; (2) the characteristics of participants: study population, sample size, age, and duration of follow-up; and (3) the events and outcomes, the adjusted HR with 95% CI of the PRISm, statistical models, covariates adjusted in the multivariable analysis, subgroup analyses, and sensitivity analyses. When studies had several adjustment models, we extracted the models reported in the abstract.

### Quality assessment

The Strengthening the Reporting of Observational Studies in Epidemiology checklist was used when assessing the eligibility of reports [13]. The Newcastle–Ottawa scale (NOS) was used to assess the quality of the cohort studies included in this review, which was a validated scale for non-randomized studies in three areas: the selection of exposed and unexposed participants, the comparability of the groups, and the assessment of the outcome [14, 15]. This scale awards a maximum of 9 points to each study: 4 points for the selection of participants and measurement of exposure, 2 points for the comparability of cohorts on the basis of the design or analysis, and 3 points for the assessment of outcomes and adequacy of follow-up. 0–3, 4–6, and 7–9 were assigned to low, moderate, and high quality of studies, respectively. For all outcome measures, an assessment of the overall quality of evidence based on the Grading of Recommendations Assessment, Development, and Evaluation (GRADE) method [16] was used across five domains, which included bias risk, inconsistency, indirectness, imprecision, and publication bias. Depending on the quality of evidence, there are four categories: high, moderate, low, and very low.

### Statistical analysis (synthesis)

The inverse variance method with adjusted HRs was used to determine the association between PRISm and all-cause mortality, CVD mortality, and respiratory-related mortality. HR estimates and summary estimates were displayed graphically in forest plots. Inter-study heterogeneity was quantified with the *I*^2^ statistic and Q test, and the overall effect was determined with the *Z*-test [17, 18]. A random-effects model was used to pool the results of the included studies. Fixed-effects model was also reported. Potential publication bias was assessed by the application of Egger’ test and Begg’s test at the *P* < 0.05 level of significance [19, 20]. If potential publication bias was indicated, a trim-fill analysis was applied to evaluate the number of missing studies, and recalculation of the pooled relative risk was done after addition of those missing hypothetical studies.

Considering the moderators of efficacy and potential heterogeneity, univariate meta-regression and subgroup analyses were conducted. We examined all potential moderators including mean age, percentage female, percentage current smokers, mean/median pack-years, body mass index, pre- or post-bronchodilation, follow-up years, and study years. Two subgroup analysis was performed: (1) smoking population [12, 21] and (2) six studies using pre-bronchodilation spirometry test to define the PRISm. Next, three sensitivity analyses were conducted to explore the robustness of the association: (1) recalculated the pooled effect estimates by omitting one study at a time to assess the robustness of the results and the influence of individual studies on heterogeneity, (2) extracted HR of baseline lung function categories defined by the lower limit of normal (LLN) criterion, and (3) accumulation analysis was conducted in chronological order of publication. Stata 16.0 were used for all statistical analyses including publication bias. Statistical significance was assumed at *P* < 0.05 using 2-tailed tests.

## Results

### Study selection and characteristics

Our search strategy retrieved 6536 potentially relevant articles. After excluding 2863 duplicates, 1914 irrelevant records, 1411 conference abstract, letters, editorials, comment, or correction, 117 reviews, 88 case reports, and 18 animal-related studies, 133 met the criteria for full-text review. Furthermore, 123 records were excluded due to the following reasons: no relevant exposure and outcome and no exact estimates and other reasons (Fig. 1). Finally, 10 articles including 8 studies with 420,218 general individuals and 2 subgroup population (smokers) were included in this meta-analysis. Of these eight studies analyzed, seven were prospective cohort studies, and 1 was a retrospective cohort study [6]. Two articles were from the same cohort (UK Biobank): one reported all-cause mortality, while the other reported CVD mortality [2, 4]. Among the studies included in the meta-analysis, three were UK cohorts [2, 4, 22], one US cohort [6], one Latin America cohort [23], one Netherlands cohort [7], one Japanese cohort [5], and one South Korea cohort [9]. We included Wan (2018) [12] and Kaaks (2022) [21] for further analysis since the participants were smokers.

The main characteristics of the selected studies were presented in Table 1. The follow-up period of the study ranged from 5 to 19 years. All studies were adjusted for age, sex, body mass index, and smoking status using multivariable Cox proportional models, and a few studies also controlled alcohol drinking, physical activity, comorbidities, and education [4, 22, 23].

**Table 1:** The characteristics of included studies in the meta-analysis

Study	Country	Cohort	Sample(n)	Population	BD	Age(yrs)	Median follow-up(yrs)	Outcome
Wan (2018) [12]^b^	US	COPDGene cohort	8800	Smoking	Post-BD	45–80^a^	5	All-cause mortality
Wijnant (2020) [7]	Netherlands	Rotterdam study	5487	General	Pre-BD	69.1	9.8 (maximum)	All-cause mortality, CVD mortality
He (2021) [22]	UK	ELSA cohort	6616	General	Pre-BD	65.8	7.7 (mean)	All-cause mortality, respiratory-related mortality, CVD mortality
Wan (2021) [6]	US	NHLBI	53,701	General	Pre-BD	53.2	19.4	All-cause mortality, respiratory-related mortality, CHD-related mortality, respiratory-related events, CHD-related events
Higbee (2022) [2]^c^	UK	UK Biobank	351,874	General	Pre-BD	40–69^a^	9	All-cause mortality
Kaaks (2022) [21]^b^	German	LUSI	1987	Smoking	Pre-BD	50–69^a^	12.1	All-cause mortality, lung cancer incidence
Washio (2022) [5]	Japan	Hisayama study	3032	General	Pre-BD	≥ 40^a^	5.3	All-cause mortality, CVD mortality, respiratory-related mortality, cancer death
Perez-Padilla (2023) [23]	Latin America	PLATINO	2942	General	Post-BD	55	5–9 (range)	All-cause mortality
Sin (2023) [9]	South Korea	KoGES	7526	General	Pre-BD	51.9	16.5	All-cause mortality, CVD mortality
Zheng (2023) [4]^c^	UK	UK Biobank	329,954	General	Pre-BD	40–69^a^	11.12	CVD mortality, MACE, MI, HF, stroke

*yrs* years, *NR* Not recorded

COPDGene cohort, a cohort study; Rotterdam study, a population-based prospective cohort; ELSA cohort, the English Longitudinal Study of Aging; *NHLBI* National Heart Lung and Blood Institute Pooled Cohorts; UK Biobank, a nationwide cohort; *LUSI* the German Lung Cancer Screening Intervention study; Hisayama study, a population-based prospective cohort study; *PLATINO* the Proyecto Latinoamericano de Investigación en Obstrucción Pulmonary, *KoGES* the Korean Genome and Epidemiology Study Ansan and Ansung study. *BD* Bronchodilation, *CVD* Cardiovascular disease; all-cause mortality, deaths from any causes; CVD mortality, deaths due to cardiovascular disease; respiratory-related mortality, deaths due to respiratory disease; *MACE* Major adverse cardiovascular event, *MI* Myocardial infarction, *HF* Heart failure, *CHD* Coronary heart disease; respiratory-related events, respiratory-related hospitalizations and mortality; CHD-related events, CHD-related hospitalizations and mortality

^a^The entry criteria for study

^b^These two studies were not included in the main analysis due to the smoking population but for the subgroup analysis

^c^Both Zheng (2023) and Higbee (2022) were from the same cohort (UK Biobank cohort). They reported on all-cause mortality and CVD mortality separately

### Risk-of-bias results

Two reviewers completed the NOS system, and the quality scores was presented in Table 2. All studies were rated as moderate or high quality. The study was rated of medium quality for three reasons: (1) the cardiovascular population was not excluded at baseline, (2) no median follow-up was recorded, and (3) lack of information on the rate of loss of follow-up [7].

**Table 2:** Quality assessment of observational studies included in the meta-analysis assessed by the Newcastle–Ottawa scale

Study	Selection				Comparability		Outcome			Score
	Representativeness	Selection of non-exposed	Ascertainment of exposure	Outcome not present at start	Comparability on most important factors	Comparability on other risk factors	Assessment of outcome	Long enough follow-up (median ≥ 5 years)	Adequacy (completeness) of follow-up	
Wan (2018) [12]	0	1	1	0	1	1	0	1	0	5
Wijnant (2020) [7]	1	1	1	0	1	1	1	0	0	6
He (2021) [22]	1	1	1	0	1	1	1	1	0	7
Wan (2021) [6]	1	1	1	0	1	1	1	1	0	7
Higbee (2022) [2]	1	1	1	0	1	1	1	1	0	7
Washio (2022) [5]	1	1	1	0	1	1	1	1	0	7
Kaaks (2022) [21]	0	1	1	0	1	1	1	1	0	6
Perez-Padilla (2023) [23]	1	1	1	0	1	1	1	0	0	6
Zheng (2023) [4]	1	1	1	1	1	1	1	1	0	8
Sin (2023) [9]	1	1	1	0	1	1	1	1	0	7

0–3 were low quality studies, 4–6 were moderate quality studies, 7–9 were high-quality studies

### Primary outcomes

**Fig. 2:**
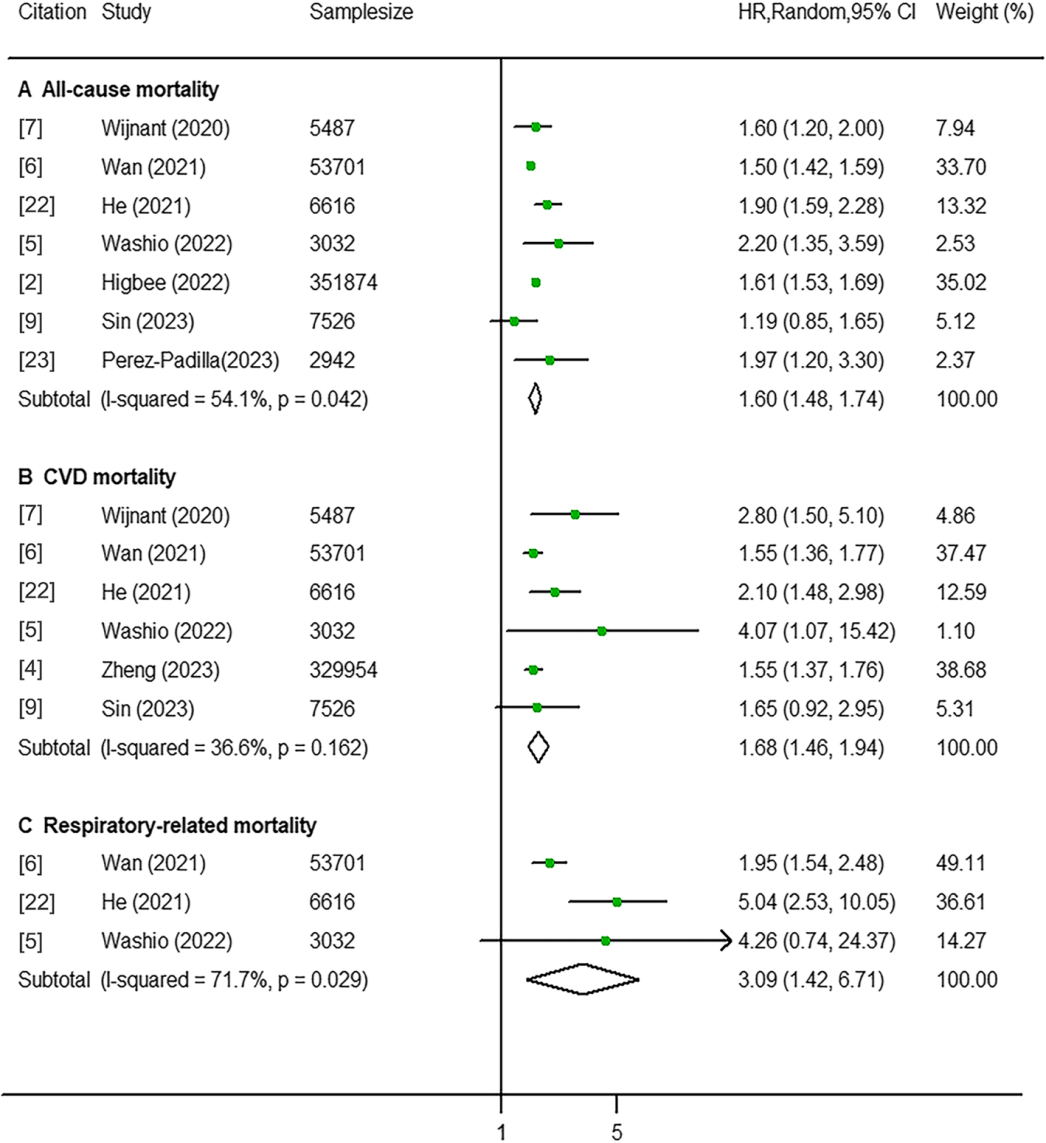
Meta-analysis of the effects of PRISm on outcomes

Our main analysis included studies involving the general population at baseline [2, 4–7, 9, 22, 23]. Fig. 2 shows the forest plot for the results among all included studies, and the details are presented in Additional file 1: Table S3 and Table S4. Compared with individuals with normal spirometry, those with PRISm had higher risks of all-cause mortality (*HR*, 1.60; 95% *CI*, 1.48–1.74), CVD mortality (*HR*, 1.68; 95% *CI*, 1.46–1.94), and respiratory-related mortality (*HR*, 3.09; 95% *CI*, 1.42–6.71) based on random effect (with *I*^2^ = 54.1%, 36.6%, and 71.7%). Additionally, the results of both fixed and random effects were similar, as Additional file 1: Table S5. Due to heterogeneity across the included studies in the meta-analysis, random-effects univariate meta-regression presented that smoking pack-years were significantly associated with all-cause mortality (Table 3). One study reported the results of specific CVD diseases, MI (*HR*, 1.12; 95% *CI*, 1.01–1.25), HF (*HR*, 1.88; 95% *CI*, 1.72–2.05), stroke (*HR*, 1.26; 95% *CI*, 1.13–1.40), and MACE (*HR*, 1.26; 95% *CI*, 1.17–1.35) [4].

**Table 3:** Results of meta-regression

	Coefficient (95% CI)	Standard error	*p*-value	Adj. R-squared
Mean age	1.008 (0.973–1.045)	0.015	0.595	− 30.15%
Mean BMI	1.004 (0.927–1.088)	0.033	0.901	− 40.77%
Female percentage (%)	0.994 (0.965–1.023)	0.012	0.617	− 2.00%
Current smoker percentage (%)	1.004 (0.996–1.012)	0.003	0.285	− 13.65%
Smoking pack-years	1.013 (1.001–1.024)	0.004	**0.038**	97.54%
Follow-up years	0.795 (0.627–1.007)	0.077	0.055	89.52%
Pre- or post-bronchodilation	1.218 (0.829–1.788)	0.198	0.265	31.99%
Study year	0.962 (0.866–1.070)	0.043	0.420	4.94%

In addition, we assessed publication bias by assessing Begg’s test and Egger’s test. The result was reported for all-cause mortality (Begg’s test: *P* = 0.881, Egger’s test: *P* = 0.71), for CVD mortality (Begg’s test: *P* = 0.091, Egger’s test: *P* = 0.025), and for respiratory-related mortality (Begg’s test: *P* = 0.602, Egger’s test: *P* = 0.408). For CVD mortality, a potential publication bias was detected. We conducted a trim-fill test for it, and the effect estimate was (1.56; 95% *CI*, 1.32–1.84; *P* < 0.05). The result is still statistically significant (Additional file 1, Figure S1).

### Assessment of evidence

The certainty of evidence for all assessed outcomes was considered “moderate” due to the observational study design and downgrades resulting from publication bias as evaluated by the GRADE method (Additional file 1, Table S6).

### Further analysis

Subgroup analysis showed that participants who were past and current smokers had a significant harmful effect (*HR*, 2.11; 95% *CI*, 1.74–2.54) on all-cause mortality with nonsignificant heterogeneity (*I*^2^ = 0, *P* = 0.54) (Additional file 1, Figure S2), which is higher than the general population [12, 21]. The pooled HR was 1.59 (95% *CI*, 1.47–1.73) for all-cause mortality using pre-bronchodilation spirometry (Additional file 1, Figure S3). Three sensitivity analyses were also performed to estimate the stability of the results.

1. A removal of one study from the effect estimate resulted in no significant change in the effect estimate, suggesting that the results were relatively stable and reliable (Additional file 1, Figure S4).
2. When using LLN thresholds to define PRISm, the pooled HR was 1.56 (95% *CI*, 1.27–1.90) for all-cause mortality, 1.56 (95% *CI*, 1.41–1.71) for CVD mortality, and 1.51 (95% *CI*, 1.29–1.78) for respiratory-related mortality. The results were consistent with the main analysis, indicating that the results are robust (Additional file 1, Figure S5).
3. After accumulation analysis conducted in chronological order, HR point estimates were stable in all-cause mortality, CVD mortality, and respiratory-related mortality (Additional file 1, Figure S6).

## Discussion

This systematic review and meta-analysis estimated the impacts of PRISm on mortality by pooling data collected from multiple studies, for all-cause mortality, CVD mortality, and respiratory-related mortality. The analysis helped to address two critical issues in the study. Firstly, the spirometric restrictive pattern defined as PRISm is a heterogeneous and unstable population in terms of their longitudinal transition to different lung function categories over time. This concept was introduced recently, and limited information is available on this topic in population-based studies. Secondly, mortality, which is more easily accessible, is thought to be reported with less bias than morbidity. In this meta-analysis, the prevalence of PRISm results was 11%. Most importantly, individuals with PRISm at baseline were associated with increased risk of all-cause mortality, CVD mortality, and respiratory-related mortality, which was consistent with several sensitivity analyses. Notably, the magnitudes of the association tended to be stronger in smokers with PRISm results than in general participants.

In population-based studies conducted in different countries and regions, the prevalence of PRISm in the included studies fluctuated between 5 and 17% [3–7, 23–26]. However, in one study conducted among the hospital-based pulmonary function testing centers, the prevalence of PRISm exceeded 20% [27], and in two studies conducted among the smoking population, the prevalence was 15.7% and 12.5% respectively [12, 21].

This study incorporated original studies that used all-cause mortality, CVD mortality, or respiratory-related mortality as their endpoint [2, 4–7, 9, 22]. After adjusting for confounders such as demographics, medical history, lifestyle behaviors, and socioeconomic factors, these studies indicated that PRISm is an independent risk factor for both all-cause mortality and CVD mortality, with HR point estimates ranging from 1.19 to 2.20 (for all-cause mortality) and 1.55–4.07 (for CVD mortality). One study with a relatively small sample size indicated an increasing trend in the risk of all-cause mortality and CVD mortality in the PRISm population, but the results were not statistically significant [9]. There were three original studies with respiratory-related death as the endpoint, two of which suggested an independent association between PRISm and respiratory-related death (HR = 1.95 and 5.04) [6, 22]. Although one study had a large HR point estimate (4.26), the result was not statistically significant, possibly due to insufficient statistical power from a small sample size [5]. The results of our meta-analysis showed that baseline PRISm exposure was significantly associated with an increased risk of all-cause mortality, CVD mortality, and respiratory-related mortality, and these associations remained robust in sensitivity analyses. These results reinforced the reliability of the association between PRISm and the three mortality-related outcomes. Moreover, since most of the original studies came from a single country or region, it cannot be ruled out that the differences in effect size among these studies may be due to variations in race, environment, culture, and socioeconomic factors. Thus, this study aimed to provide a global estimate of the health risks related to PRISm exposure. The study also focused on health outcomes such as stroke, MI, and HF. However, as there was only one original study corresponding to each of these outcomes [4], we merely summarized their results without conducting a meta-analysis. Further exploration is needed regarding the association between PRISm and these outcomes.

PRISm represents a heterogeneous group of populations, potentially combining elements of both obstructive and restrictive lung disease. This includes individuals with early-stage chronic obstructive pulmonary disease (COPD), asthma, obesity-related lung restriction, and interstitial lung disease [3]. Consequently, it is inadvisable to directly employ the concept of PRISm as a clinical diagnostic criterion or for therapeutic guidance. Nonetheless, given the accessibility of simplified lung function tests and the clear association of this pulmonary phenotype with adverse outcomes, PRISm presents significant value for public health screening. Patients flagged for PRISm during pulmonary function screening should undergo comprehensive evaluations, including total lung capacity, pulmonary diffusion function, and radiographic examinations of the lungs to refine diagnosis and inform treatment strategies. Moreover, univariate meta-regression suggested that smoking pack-years is a significant source of the heterogeneity between the included studies. Confining the definition of PRISm solely to smokers could lead to a population more characterized by obstructive ventilatory impairments, thereby enhancing its homogeneity [28]. Our research suggests that the all-cause mortality risk is higher for PRISm populations defined based on smokers compared to those defined from the general population. However, subgroup analyses from earlier studies have not shown significant variations in the relationship between PRISm and overall or CVD mortality across subgroups stratified by different smoking statuses [4, 6]. Thus, the necessity of confining PRISm to smokers remains controversial. A potential heterogeneity factor includes duration of follow-up and pre- or post-bronchodilation spirometry.

Investigations are being conducted to examine the radiographic characteristics of PRISm. One of the main feature distinguishing PRISm from normal condition is the presence of inflammation and remodeling in the respiratory bronchioles and peripheral alveolar tissue, resulting in functional small airway disease (fSAD), along with a reduction in pulmonary small vessels [29]. Additionally, the primary difference between PRISm and COPD is that an individual with PRISm has not yet experienced parenchymal damage or significant emphysematous changes in the lungs [30]. The current understanding on the pathological and anatomical characteristics of PRISm may not be enough, and, therefore, more in-depth research is desired. As for the pathological and pathophysiological mechanisms of PRISm, it is currently postulated that a combination of airway remodeling, lung parenchymal damage, and systemic inflammation may play a role [4]. Chronic inflammation can cause changes in the airway structure, which may result in reduced airflow and lung volume. Damaged parenchymal tissue can further contribute to impaired lung function by decreasing elastic recoil. Additionally, excess adipose tissue in obesity-related lung restriction can compress the lungs mechanically, leading to reduced lung volumes [29]. Furthermore, there may be systemic inflammation or comorbid conditions that contribute to the phenotype [3], but this needs further research. However, these mechanisms remain largely hypothetical, and more studies are needed to fully understand the underlying disease processes in PRISm.

The strengths of the current evaluation include the following: (1) the use of only observational cohort studies with a mean quality score of 6.9 for 8 included studies (6.6 for 10 included studies) and at least 5 years follow-up, hence ensuring temporality in the association; (2) it combined multiple studies from a range of geographical locations, containing a large number of individuals; (3) assessment of the risk of bias for each individual study and the certainty of the evidence using well-established tools; and (4) several sensitivity analyses were conducted to test the robustness of the association.

Most of the limitations are inherent to the studies, not the methodology. First, the definition of PRISm is inconsistent (pre- and post-bronchodilation) across included studies and other substantial heterogeneity in the study population in terms of age, smoking status, lung function test, and severity of the disease. Second, given the varying degree of adjustment across studies, we could not evaluate the impact of a uniform approach to statistical adjustment. For example, whereas some studies adjusted important confounding factors, such as pack-year in the model, others did not adjust it. This did not enable transformation into consistent comparisons. Hence, a fully adjusted model in each study was used to synthesize the data. Third, due to limited studies and reported data, subgroup analysis was not conducted. Moreover, the comparisons between smokers and the general population may have been biased as they were from different studies. Fourth, the use of observational study designs with PRISm findings assessed at baseline may have led to potential biases such as reverse causation and regression dilution. A meta-analysis of individual participant data with objective measures of lung function and their repeat measures may better quantify the association between lung function and mortality risk. Finally, searches were not conducted in some databases (Scopus) due to access restrictions, potentially resulting in incomplete information and publication bias.

Furthermore, the association between PRISm and adverse health outcomes is well established. Based on this systematic review, only a few studies have investigated the association between PRISm and cardiovascular outcomes, indicating that further research to investigate the relationship between PRISm and different clinical outcomes is needed in the future. The included studies in this analysis were single-country study; considering the geographically variations in PRISm, a global multicenter study is needed to assess the association among different region and ethnicity. In addition, the definition of PRISm has been revised to smokers after bronchodilation in the recent ATS/ERS guidelines published in 2023. However, among the 10 studies included in our analysis, only 2 were conducted among smokers, and 2 utilized post-bronchodilation spirometry. Further study is needed to test the variation in the definition and to align with the latest PRISm definition [1, 28]. However, our current understanding of the etiology and progression mechanisms of PRISm remains significantly limited, and effective prevention and treatment strategies are lacking. Future research is required to elucidate the mechanisms of PRISm and to identify effective interventions to reduce the incidence of PRISm and its associated health risks.

## Conclusions

Individuals with PRISm have a higher risk of mortality compared to those with normal lung function in the general population. The association between PRISm and all-cause mortality is higher in the smoking population, suggesting that the smoking population should be the main target population for intervention. Given the high prevalence and high risk of mortality-related outcomes of PRISm, further studies are needed to investigate the detailed mechanisms and appropriate treatment measures.

### Supplementary Information

Additional file: Table S1. Search strategy in PubMed, EMBASE, and Web of Science. Table S2. PPRISMA 2020 Checklist. Table S3. Summary of the association between PRISm and mortality. Table S4. Statistical analysis model details for included studies. Table S5. Hazard Ratio estimates using fixed-effects model. Table S6. GRADE assessment. Figure S1. Trim-fill test on the effect of PRISm on CVD mortality. Figure S2. Subgroup analysis of the effect of PRISm on mortality among smokers. Figure S3. Analyses for pre- bronchodilation. Figure S4. Leave-one-out analyses. Figure S5. Sensitivity analyses of Global Lung Initiative definitions for FEV1 and FVC with LLN thresholds. Figure S6. Accumulation analyses.

## Data Availability

All data relevant to the study are included in the article or uploaded as online supplemental information.

## Abbreviations

ATS: American Thoracic Society
CHD: Coronary heart disease
CI: Confidence interval
CVD: Cardiovascular disease
ERS: The European Respiratory Society
FEV1: The forced expiratory volume in one second
fSAD: Functional small airway disease
FVC: The forced vital capacity
GOLD: Global Initiative for Chronic Obstructive Lung Disease
HF: Heart failure
HR: Hazard ratio
LLN: Lower limit of normal
MACE: Major adverse cardiovascular event
MI: Myocardial infarction
NOS: Newcastle-Ottawa scale
PRISm: Preserved ratio impaired spirometry
