# Supplementary material for: Association of preserved ratio impaired spirometry with mortality and cardiovascular diseases: a systematic review and meta-analysis
Source: Syst Rev. 2024 Jul 6;13:171. doi: 10.1186/s13643-024-02549-6 (PMC11227213; doi:10.1186/s13643-024-02549-6)
Supplement: Supplementary file 1 — Additional file: Table S1. Search strategy in PubMed, EMBASE, and Web of Science. Table S2. PPRISMA 2020 Checklist. Table S3. Summary of the association between PRISm and mortality. Table S4. Statistical analysis model details for included studies. Table S5. Hazard Ratio estimates using fixed-effects model. Table S6. GRADE assessment. Figure S1. Trim-fill test on the effect of PRISm on CVD mortality. Figure S2. Subgroup analysis of the effect of PRISm on mortality among smokers. Figure S3. Analyses for pre- bronchodilation. Figure S4. Leave-one-out analyses. Figure S5. Sensitivity analyses of Global Lung Initiative definitions for FEV1 and FVC with LLN thresholds. Figure S6. Accumulation analyses. [file 13643_2024_2549_MOESM1_ESM.docx]

**SUPPLEMENTARY INFORMATION**

**Association of preserved ratio impaired spirometry with mortality: a systematic review and meta-analysis**

**Table S1. Search strategy in PubMed, EMBASE, and Web of Science**

| **Database** | **Search strategy** | **Hits** |
| --- | --- | --- |
| PUBMED | ((((((((((((((("preserved ratio impaired spirometry"[Title/Abstract]) OR ("PRISm spirometry"[Title/Abstract])) OR ("restrictive spirometry"[Title/Abstract])) OR ("restrictive lung disease"[Title/Abstract])) OR ("restrictive pulmonary disease"[Title/Abstract])) OR ("restrictive lung patterns"[Title/Abstract])) OR ("restrictive lung defect"[Title/Abstract])) OR ("restrictive pulmonary defect"[Title/Abstract])) OR ("restrictive lung function"[Title/Abstract])) OR ("restrictive pulmonary function"[Title/Abstract])) OR ("low lung function"[Title/Abstract])) OR ("unclassified spirometry"[Title/Abstract])) OR ("non-specific spirometry"[Title/Abstract])) OR ("global initiative for chronic obstructive lung disease unclassified"[Title/Abstract])) OR ("GOLD-unclassified"[Title/Abstract])) OR ("GOLD-U"[Title/Abstract]) | 1657 (2023.09.07) |
| EMBASE | ('preserved ratio impaired spirometry':ab,ti OR 'prism spirometry':ab,ti OR 'restrictive spirometry':ab,ti OR 'restrictive lung disease':ab,ti OR 'restrictive pulmonary disease':ab,ti OR 'restrictive lung patterns':ab,ti OR 'restrictive lung defect':ab,ti OR 'restrictive pulmonary defect':ab,ti OR 'restrictive lung function':ab,ti OR 'restrictive pulmonary function':ab,ti OR 'low lung function':ab,ti OR 'unclassified spirometry':ab,ti OR 'non-specific spirometry':ab,ti OR 'global initiative for chronic obstructive lung disease unclassified':ab,ti OR 'gold-unclassified':ab,ti OR 'gold-u':ab,ti) AND [embase]/lim | 3153  (2023.09.07) |
| Web of Science | (((((((((((((((((((((((((((((((TI=(“preserved ratio impaired spirometry”)) OR AB=(“preserved ratio impaired spirometry”)) OR TI=(“PRISm spirometry”)) OR AB=(“PRISm spirometry”)) OR TI=(“restrictive spirometry”)) OR AB=(“restrictive spirometry”)) OR AB=(“restrictive lung disease”)) OR TI=(“restrictive lung disease”)) OR TI=(“restrictive pulmonary disease”)) OR AB=(“restrictive pulmonary disease”)) OR AB=(“restrictive lung patterns”)) OR TI=(“restrictive lung patterns”)) OR TI=(“restrictive lung defect”)) OR AB=(“restrictive lung defect”)) OR AB=(“restrictive pulmonary defect” )) OR TI=(“restrictive pulmonary defect” )) OR TI=(“restrictive lung function”)) OR AB=(“restrictive lung function”)) OR AB=(“restrictive pulmonary function”)) OR TI=(“restrictive pulmonary function”)) OR TI=(“low lung function”)) OR AB=(“low lung function”)) OR AB=(“unclassified spirometry”)) OR TI=(“unclassified spirometry”)) OR TI=(“non-specific spirometry”)) OR AB=(“non-specific spirometry”)) OR AB=(“global initiative for chronic obstructive lung disease unclassified”)) OR TI=(“global initiative for chronic obstructive lung disease unclassified”)) OR TI=(“GOLD-unclassified”)) OR AB=(“GOLD-unclassified”)) OR AB=(“GOLD-U”)) OR TI=(“GOLD-U”) | 1726  (2023.09.07) |

TI: Title; AB: Abstract.

**Table S2. PPRISMA 2020 Checklist**

| **Section and Topic** | **Item #** | **Checklist item** | **Location where item is reported** |
| --- | --- | --- | --- |
| **TITLE** | | |  |
| Title | 1 | Identify the report as a systematic review. | 1 |
| **ABSTRACT** | | |  |
| Abstract | 2 | See the PRISMA 2020 for Abstracts checklist. | 1 |
| **INTRODUCTION** | | |  |
| Rationale | 3 | Describe the rationale for the review in the context of existing knowledge. | 2 |
| Objectives | 4 | Provide an explicit statement of the objective(s) or question(s) the review addresses. | 2 |
| **METHODS** | | |  |
| Eligibility criteria | 5 | Specify the inclusion and exclusion criteria for the review and how studies were grouped for the syntheses. | 2 |
| Information sources | 6 | Specify all databases, registers, websites, organisations, reference lists and other sources searched or consulted to identify studies. Specify the date when each source was last searched or consulted. | 2 |
| Search strategy | 7 | Present the full search strategies for all databases, registers and websites, including any filters and limits used. | 2 |
| Selection process | 8 | Specify the methods used to decide whether a study met the inclusion criteria of the review, including how many reviewers screened each record and each report retrieved, whether they worked independently, and if applicable, details of automation tools used in the process. | 2-3 |
| Data collection process | 9 | Specify the methods used to collect data from reports, including how many reviewers collected data from each report, whether they worked independently, any processes for obtaining or confirming data from study investigators, and if applicable, details of automation tools used in the process. | 2-4 |
| Data items | 10a | List and define all outcomes for which data were sought. Specify whether all results that were compatible with each outcome domain in each study were sought (e.g. for all measures, time points, analyses), and if not, the methods used to decide which results to collect. | 2-3 |
|  | 10b | List and define all other variables for which data were sought (e.g. participant and intervention characteristics, funding sources). Describe any assumptions made about any missing or unclear information. | 2-4 |
| Study risk of bias assessment | 11 | Specify the methods used to assess risk of bias in the included studies, including details of the tool(s) used, how many reviewers assessed each study and whether they worked independently, and if applicable, details of automation tools used in the process. | 4 |
| Effect measures | 12 | Specify for each outcome the effect measure(s) (e.g. risk ratio, mean difference) used in the synthesis or presentation of results. | 4 |
| Synthesis methods | 13a | Describe the processes used to decide which studies were eligible for each synthesis (e.g. tabulating the study intervention characteristics and comparing against the planned groups for each synthesis (item #5)). | 4 |
|  | 13b | Describe any methods required to prepare the data for presentation or synthesis, such as handling of missing summary statistics, or data conversions. | 4 |
|  | 13c | Describe any methods used to tabulate or visually display results of individual studies and syntheses. | 4 |
|  | 13d | Describe any methods used to synthesize results and provide a rationale for the choice(s). If meta-analysis was performed, describe the model(s), method(s) to identify the presence and extent of statistical heterogeneity, and software package(s) used. | 4 |
|  | 13e | Describe any methods used to explore possible causes of heterogeneity among study results (e.g. subgroup analysis, meta-regression). | 4 |
|  | 13f | Describe any sensitivity analyses conducted to assess robustness of the synthesized results. | 4 |
| Reporting bias assessment | 14 | Describe any methods used to assess risk of bias due to missing results in a synthesis (arising from reporting biases). | 4 |
| Certainty assessment | 15 | Describe any methods used to assess certainty (or confidence) in the body of evidence for an outcome. | 4 |
| **RESULTS** | | |  |
| Study selection | 16a | Describe the results of the search and selection process, from the number of records identified in the search to the number of studies included in the review, ideally using a flow diagram. | 4 |
|  | 16b | Cite studies that might appear to meet the inclusion criteria, but which were excluded, and explain why they were excluded. | 4 |
| Study characteristics | 17 | Cite each included study and present its characteristics. | 4 |
| Risk of bias in studies | 18 | Present assessments of risk of bias for each included study. | 4-5 |
| Results of individual studies | 19 | For all outcomes, present, for each study: (a) summary statistics for each group (where appropriate) and (b) an effect estimate and its precision (e.g. confidence/credible interval), ideally using structured tables or plots. | 5 |
| Results of syntheses | 20a | For each synthesis, briefly summarise the characteristics and risk of bias among contributing studies. | 5-6 |
|  | 20b | Present results of all statistical syntheses conducted. If meta-analysis was done, present for each the summary estimate and its precision (e.g. confidence/credible interval) and measures of statistical heterogeneity. If comparing groups, describe the direction of the effect. | 5-7 |
|  | 20c | Present results of all investigations of possible causes of heterogeneity among study results. | 5-7 |
|  | 20d | Present results of all sensitivity analyses conducted to assess the robustness of the synthesized results. | 5-7 |
| Reporting biases | 21 | Present assessments of risk of bias due to missing results (arising from reporting biases) for each synthesis assessed. | 4-7 |
| Certainty of evidence | 22 | Present assessments of certainty (or confidence) in the body of evidence for each outcome assessed. | 5-7 |
| **DISCUSSION** | | |  |
| Discussion | 23a | Provide a general interpretation of the results in the context of other evidence. | 8-9 |
|  | 23b | Discuss any limitations of the evidence included in the review. | 9 |
|  | 23c | Discuss any limitations of the review processes used. | 9 |
|  | 23d | Discuss implications of the results for practice, policy, and future research. | 9-10 |
| **OTHER INFORMATION** | | |  |
| Registration and protocol | 24a | Provide registration information for the review, including register name and registration number, or state that the review was not registered. | 1 |
|  | 24b | Indicate where the review protocol can be accessed, or state that a protocol was not prepared. | 1 |
|  | 24c | Describe and explain any amendments to information provided at registration or in the protocol. | 10 |
| Support | 25 | Describe sources of financial or non-financial support for the review, and the role of the funders or sponsors in the review. | 10 |
| Competing interests | 26 | Declare any competing interests of review authors. | 10 |
| Availability of data, code and other materials | 27 | Report which of the following are publicly available and where they can be found: template data collection forms; data extracted from included studies; data used for all analyses; analytic code; any other materials used in the review. | 10 |

**Table S3. Summary of the association between PRISm and mortality**

| **First author, publication year, country** | **Reference group** | **All-cause mortality** | | **CVD mortality** | | **Respiratory-related mortality** | |
| --- | --- | --- | --- | --- | --- | --- | --- |
|  |  | **Events(n)** | **HR (95%CI)** | **Events(n)** | **HR (95%CI)** | **Events(n)** | **HR (95%CI)** |
| Wan (2018), US^*^ | Normal | NR | 2.02 (1.60, 2.54) | NR | NR | NR | NR |
| Wijnant (2020), Netherlands | Normal | 692 | 1.6 (1.2, 2.0) | NR | 2.8 (1.5, 5.1) | NR | NR |
| He (2021), UK | Normal | 874 | 1.90 (1.59, 2.28) | 203 | 2.10 (1.48, 2.98) | 83 | 5.04 (2.53, 10.05) |
| Wan (2021), US | Normal | 15661 | 1.50 (1.42, 1.59) | 2352 | 1.55 (1.36, 1.77) | 1100 | 1.95 (1.54, 2.48) |
| Higbee (2022), UK^**^ | Normal | 12810 | 1.61 (1.53, 1.69) | NR | NR | NR | NR |
| Kaaks (2022), German^*^ | Normal | 54 | 2.29 (1.65, 3.19) | NR | NR | NR | NR |
| Washio (2022), Japan | Normal | 131 | 2.20 (1.35, 3.59) | 22 | 4.07 (1.07, 15.42) | 10 | 4.26 (0.74, 24.37) |
| Perez-Padilla(2023), Latin America | Normal | 301 | 1.97 (1.2, 3.3) | NR | NR | NR | NR |
| Sin (2023), South Korea | Normal | 500 | 1.19 (0.85, 1.65) | 106 | 1.65 (0.92, 2.95) | NR | NR |
| Zheng (2023), UK^**^ | Normal | NR | NR | 2,500 | 1.55 (1.37, 1.76) | NR | NR |

†The entry criteria for study.

*These two studies were included for the subgroup analysis due to the smoking population.

**Both Zheng (2023) and Higbee (2022) were from the same cohort (UK Biobank cohort). They reported on all cause mortality and CVD mortality respectively.

NR: not recorded; HR: hazard ratio; CI: confidence interval; CVD: cardiovascular disease; All-cause mortality: deaths from any causes; CVD mortality: deaths due to cardiovascular disease; Respiratory-related mortality: deaths due to respiratory disease.

**Table S4. Statistical analysis model details for included studies**

| **Citation** | **First author, publication year, country** | **Regression model** | **Full model adjustment** | **Subgroup analysis** | **Sensitivity analysis** |
| --- | --- | --- | --- | --- | --- |
| [12] | Wan (2018), US | Cox models | age, race, sex, current smoking status, cumulative smoke exposure, and BMI. | / | / |
| [7] | Wijnant (2020), Netherlands | Cox models | age, sex, BMI, current smoking and pack-years. | / | (1) excluded asthma subjects; (2) GLI definitions for FEV1 and FVC with LLN thresholds. |
| [22] | He (2021), UK | Cox models | age, sex, marital status, education level, BMI, baseline CVD and cancer, smoking status, drinking status, and physical activity level. | Sex and smoking status | (1) LLN criteria to define the lung function categories; (2) excluded subjects with asthma at baseline. |
| [6] | Wan (2021), US | Cox models | age, sex, race and ethnicity, education, body mass index, smoking status, and medical comorbidities. | Smoking status, comorbid conditions, and cohort | GLI-LLN–defined lung function categories including additional adjustment for the FEV1/FVC ratio and limiting to complete case data. |
| [2] | Higbee (2022), UK | Cox models | smoking status, BMI, age and sex. | Sex, BMI, asthma, smoking status | GLI-LLN definition of spirometry criteria, and using GOLD II-IV as the definition of obstruction. |
| [21] | Kaaks (2022), German | Cox models | age, sex, lifetime smoking duration, average cigarettes/day, time since quitting (for ex-smokers), diabetes. | / | / |
| [5] | Washio (2022), Japan | Cox models | age, sex, current smoking, smoking pack-years, and body mass index. | Smoking status | (1) GLI definitions for FEV1 and FVC with LLN thresholds; (2) nonusers of bronchodilators, nonusers of ICSs, participants with NT-proBNP, 300 pg/ml, or participants without kidney dysfunction; (3) excluded 523 participants with AFL GOLD 1–4 at baseline. |
| [23] | Perez-Padilla (2023), Latin America | Cox models | age, sex, BMl, comorbidities, cig pack-day and years at schoole. | / | / |
| [9] | Sin (2023), South Korea | Cox models | age, sex, BMI, smoking status, and comorbidities. | / | (1) LLN criteria to define the lung function categories. |
| [4] | Zheng (2023), UK | Cox models | age, sex, Townsend Deprivation Index, ethnicity, household income, height, BMI, smoking status, alcohol intake, diabetes, hyperlipidemia, high BP, and renal impairment. | Smoking status | (1) competing risks analysis with all-cause mortality as a competing event for MACE, MI, HF, and stroke; (2) Global Lung Initiative definitions for FEV1 and FVC with lower limit of normal (LLN) thresholds; (3) excluded patients with interstitial lung diseases or comorbidity, patients with missing values for covariates. |

LLN: lower limit of normal; BMI: body mass index; BP: blood pressure.

**Table S5.** **Hazard Ratio estimates using fixed-effects model**

|  |  |  |  | **Random-effects analysis** | **Fixed-effects analysis** |  |
| --- | --- | --- | --- | --- | --- | --- |
| **Outcome** | **Studies** | **Participants (n)** | **I ^2^** | **HR( 95% CI )** | **HR( 95% CI )** |  |
| All-cause mortality | 7 | 431178 | 54.10% | 1.60 ( 1.48, 1.74 ) | 1.57 ( 1.52, 1.63 ) |  |
| CVD mortality | 6 | 406316 | 36.60% | 1.68 ( 1.46, 1.94 ) | 1.61 ( 1.47, 1.75 ) |  |
| Respiratory-related mortality | 3 | 63349 | 71.70% | 3.09 ( 1.42, 6.71 ) | 2.18 ( 1.75, 2.73 ) |  |

HR: hazard ratio; CI: confidence interval; CVD: cardiovascular disease.

**Table S6. GRADE assessment**

|  | |  |  |  |  |  |  |  |  |
| --- | --- | --- | --- | --- | --- | --- | --- | --- | --- |
| **Outcome** | **Relative effect (95%CI）** | | **No of patients (studies)** | **Risk of bias** | **Inconsistency** | **Indirectness** | **Imprecision** | **Publication bias** | **Quality** |
| **All-cause mortality** | **RR 1.60**  (1.48, 1.74） | | 431178 (7 cohorts) | Not serious | Not serious | Not serious | Not serious | Serious^a^ | Moderate |
| **CVD mortality** | **RR 1.68** (1.46, 1.94) | | 406316 (6 cohorts) | Not serious | Not serious | Not serious | Not serious | Serious^a^ | Moderate |
| **Respiratory-related mortality** | **RR 3.09**  (1.42, 6.71) | | 63349 (3 cohorts) | Not serious | Not serious | Not serious | Not serious | Serious^a^ | Moderate |

GRADE: Grading of Recommendations Assessment, Development, and Evaluation; CI: confidence interval.

1. high risk of publication bias.

**Figure S1. Trim-fill test on the effect of PRISm on CVD mortality**

CVD: cardiovascular disease.

**Figure S2. Subgroup analyses of the effect of PRISm on mortality among smokers**

**
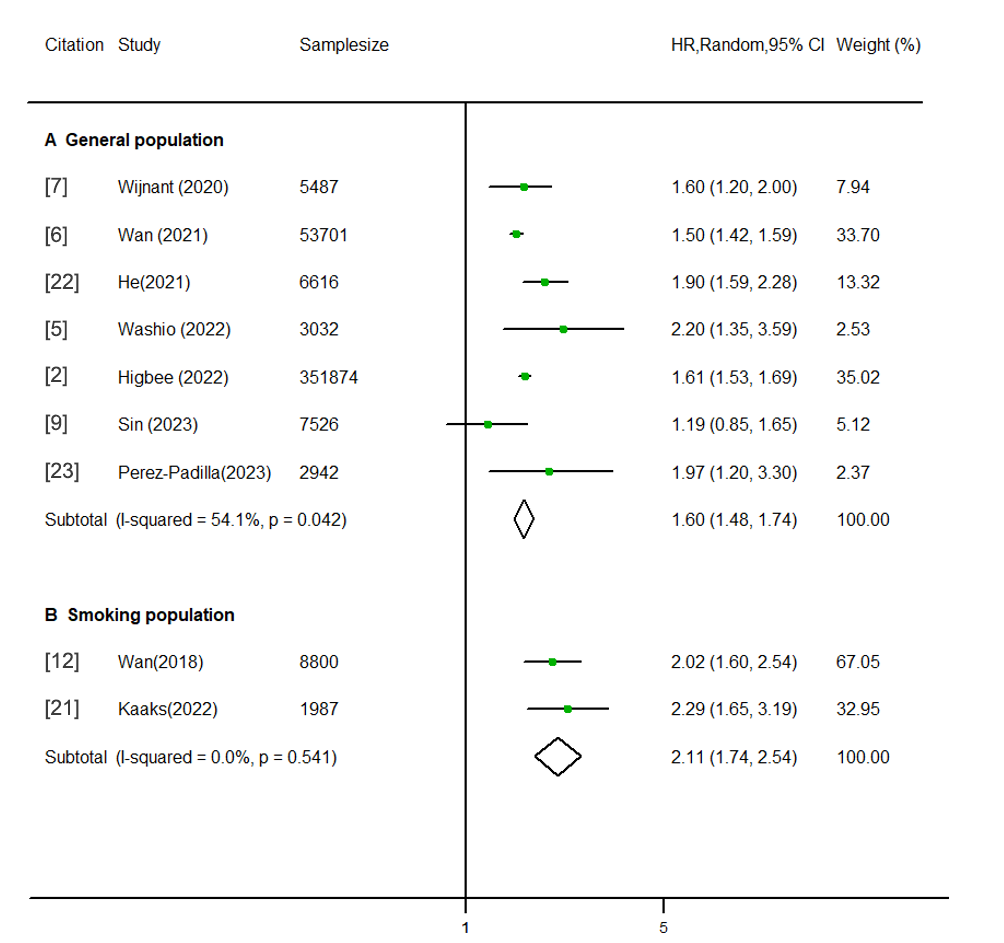
**

A: All-cause mortality among general population; B: All-cause mortality among smoking population; HR: hazard ratio; CI: confidence interval.

**Figure S3. Analyses for pre-** **bronchodilation spirometry**

**
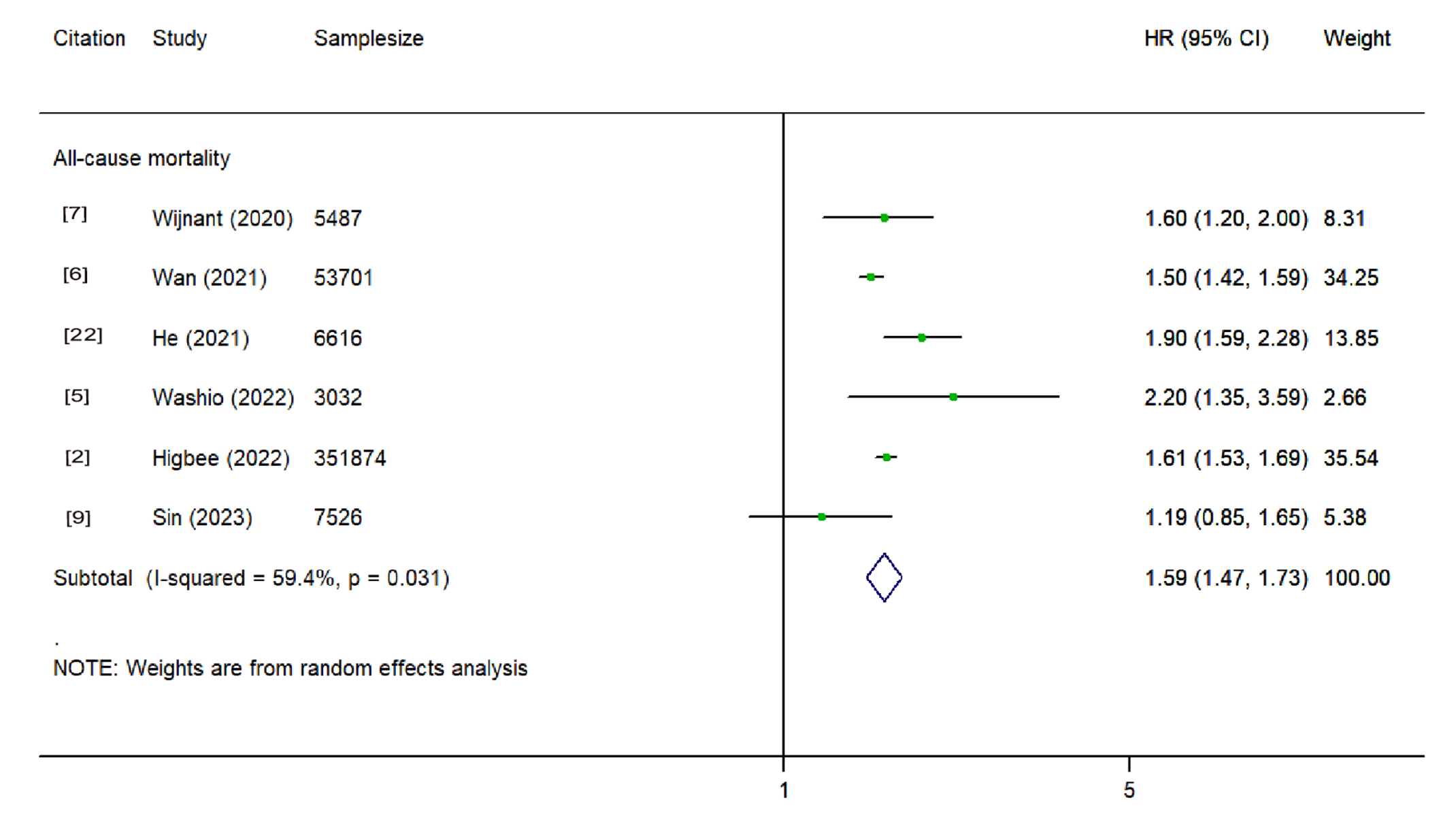
**

HR: hazard ratio; CI: confidence interval.

**Figure S4.** **Leave-one-out analyses**

**
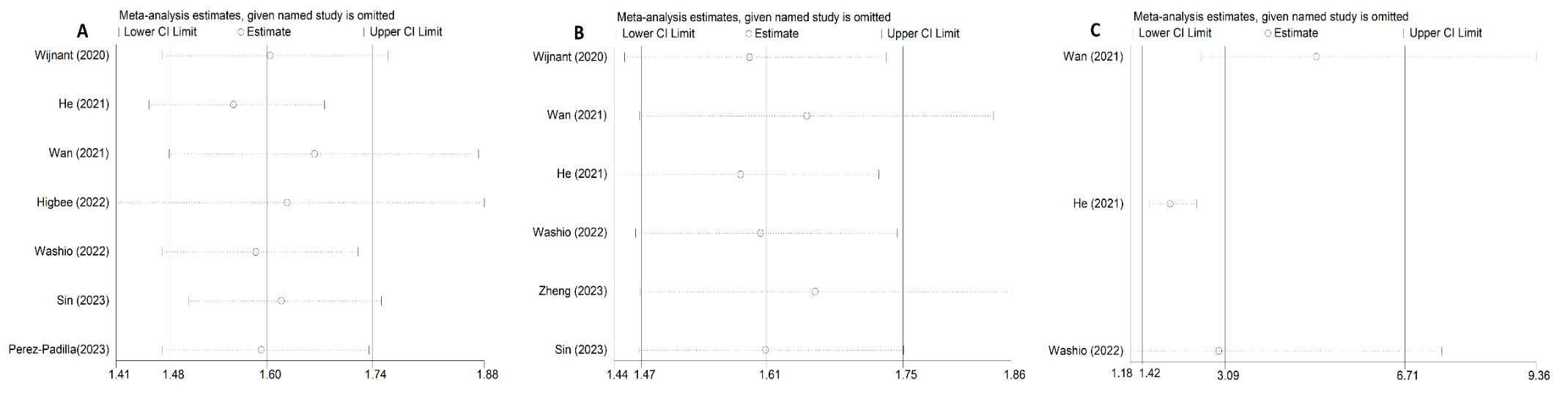
**

A: All-cause mortality; B: CVD mortality; C: Respiratory-related mortality; CVD: cardiovascular disease; CI: confidence interval.

**Figure S5. Sensitivity analyses using Global Lung Initiative definitions for FEV1 and FVC with LLN thresholds**


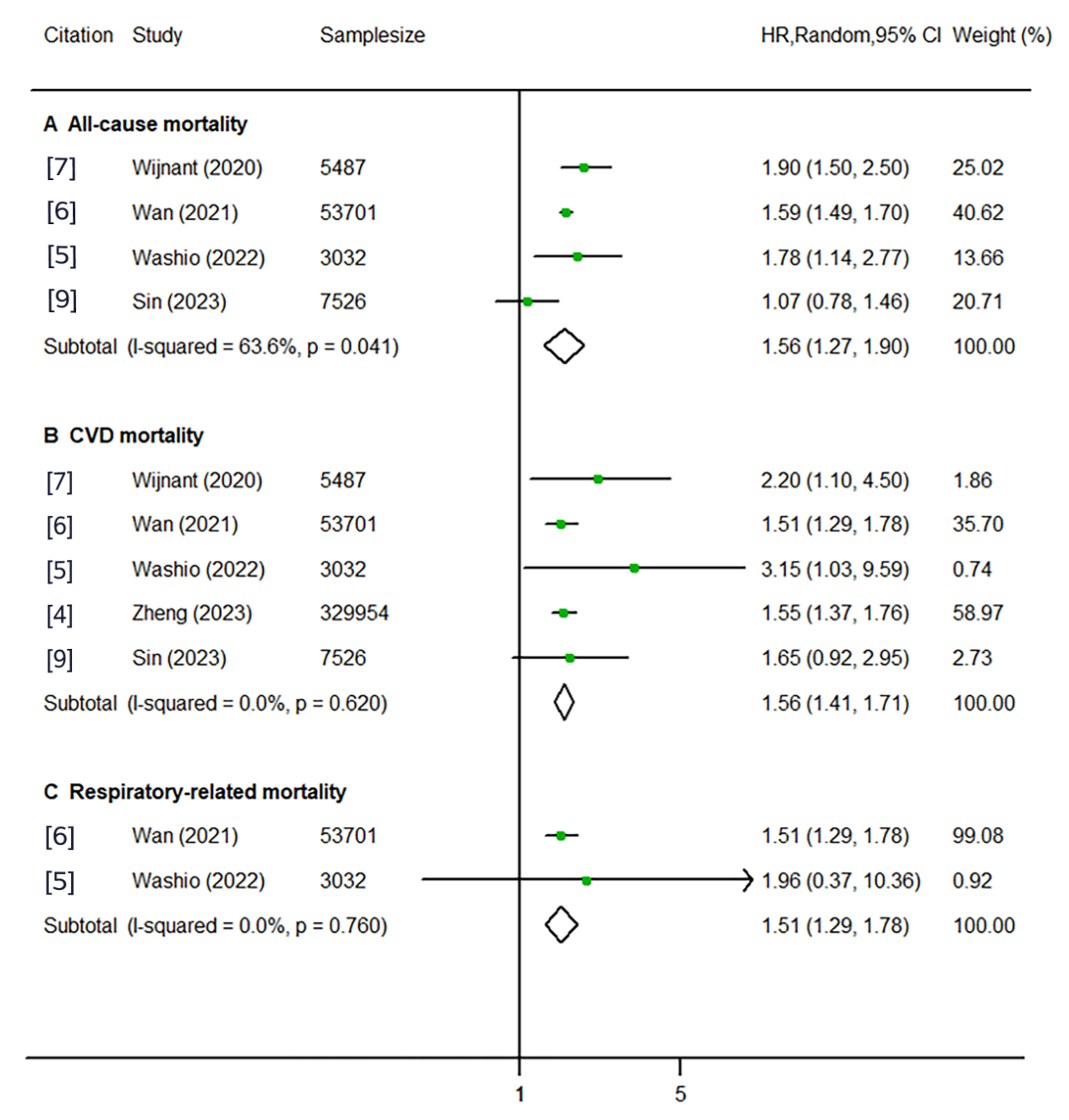


LLN: lower limit of normal; CVD: cardiovascular disease; HR: hazard ratio; CI: confidence interval.

Lung function categories were defined as, PRISm-LLN = FEV1/FVC≥LLN, FEV1<LLN; Normal Spirometry-LLN = FEV1/FVC≥LLN, FEV1≥LLN.

**Figure S6. Accumulation analyses**

**
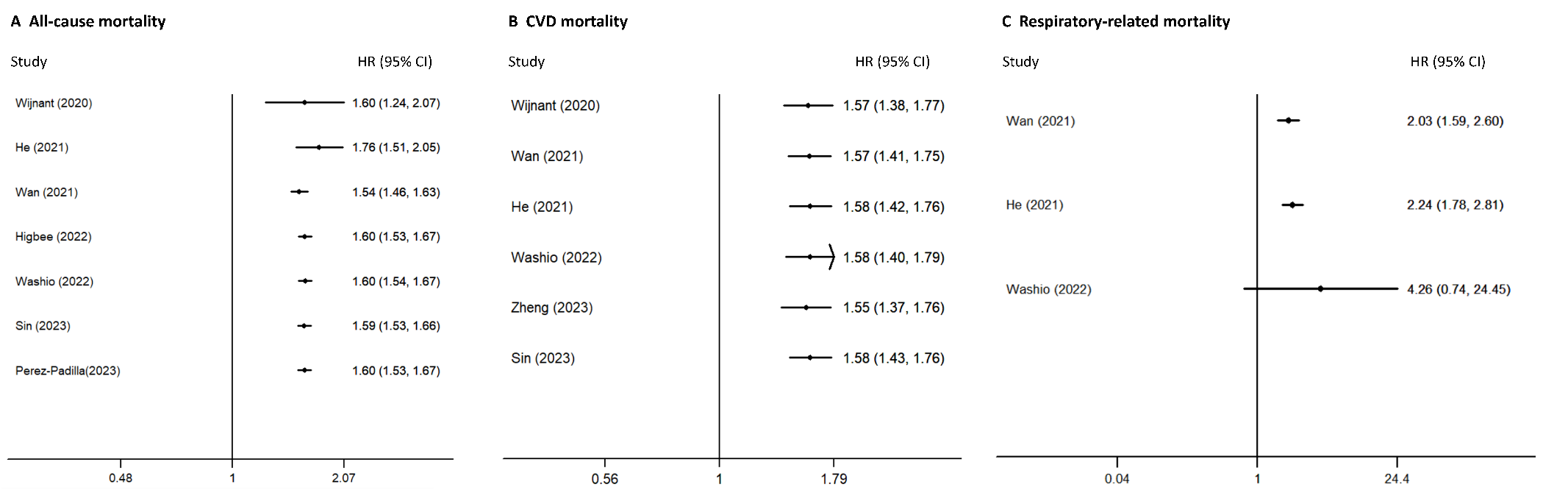
**

CVD: cardiovascular disease; HR: hazard ratio; CI: confidence interval.
